# IRWNRLPI: Integrating Random Walk and Neighborhood Regularized Logistic Matrix Factorization for lncRNA-Protein Interaction Prediction

**DOI:** 10.3389/fgene.2018.00239

**Published:** 2018-07-04

**Authors:** Qi Zhao, Yue Zhang, Huan Hu, Guofei Ren, Wen Zhang, Hongsheng Liu

**Affiliations:** ^1^School of Mathematics, Liaoning University, Shenyang, China; ^2^Research Center for Computer Simulating and Information Processing of Bio-Macromolecules of Liaoning Province, Shenyang, China; ^3^School of Life Science, Liaoning University, Shenyang, China; ^4^School of Information, Liaoning University, Shenyang, China; ^5^School of Computer, Wuhan University, Wuhan, China; ^6^Engineering Laboratory for Molecular Simulation and Designing of Drug Molecules of Liaoning, Shenyang, China

**Keywords:** lncRNA, protein, interaction prediction, random walk, neighborhood regularized logistic matrix factorization, integration method

## Abstract

Long non-coding RNA (lncRNA) plays an important role in many important biological processes and has attracted widespread attention. Although the precise functions and mechanisms for most lncRNAs are still unknown, we are certain that lncRNAs usually perform their functions by interacting with the corresponding RNA- binding proteins. For example, lncRNA-protein interactions play an important role in post transcriptional gene regulation, such as splicing, translation, signaling, and advances in complex diseases. However, experimental verification of lncRNA-protein interactions prediction is time-consuming and laborious. In this work, we propose a computational method, named IRWNRLPI, to find the potential associations between lncRNAs and proteins. IRWNRLPI integrates two algorithms, random walk and neighborhood regularized logistic matrix factorization, which can optimize a lot more than using an algorithm alone. Moreover, the method is semi-supervised and does not require negative samples. Based on the leave-one-out cross validation, we obtain the AUC of 0.9150 and the AUPR of 0.7138, demonstrating its reliable performance. In addition, by means of case study in the “Mus musculus,” many lncRNA-protein interactions which are predicted by our method can be successfully confirmed by experiments. This suggests that IRWNRLPI will be a useful bioinformatics resource in biomedical research.

## Introduction

A great quantity of studies has indicated that more than 90% of DNA is transcribed into RNA in human organism, the vast majority of which are non-coding RNA. Non-coding RNA (ncRNA) is a RNA that does not encode a protein, and plays a very broad regulatory role in many organisms' life activities. Abundant and functionally important types of non-coding RNAs include transfer RNA (tRNA) and ribosomal RNA (rRNA), and small RNAs such as microRNAs, siRNAs, piRNAs, snoRNAs, snRNAs, exRNAs, scaRNAs, and the long non-coding RNAs. Long non-coding RNA (lncRNA) refers to ncRNA longer than 200 nucleotides. LncRNA was originally considered a “noise” of genomic transcription, a byproduct of RNA polymerase II transcription, without biological function. But recent studies indicate lncRNA involves in a variety of important regulatory procedures, such as chromatin modification (Guttman et al., 2009), cell differentiation and proliferation (Wapinski and Chang, 2011), RNA progressing (Wilusz et al., 2009), and cellular apoptosis (Yu et al., 2015) and so on. These lncRNA regulation effects begin to attract widespread attention from the abnormal convey of biological cell genes. In addition, more and more experiments demonstrate that lncRNAs involve in the regulation of a variety of physiological and pathological processes, as well as the development processes of a variety of diseases including tumors (Wilusz et al., 2009; Harries, 2012; Chen and Yan, 2013; Morlando et al., 2014; Chen et al., 2015, 2016c, 2017d, 2018a,b; Yu et al., 2015; Chen and Huang, 2017b; Li et al., 2017; You et al., 2017). For instance, Gupta et al. issued an increase in the expression of lncRNA HOTAIR in primary breast tumors (Gupta et al., 2010). Along with the growth of bioinformatics, many lncRNAs have been discovered, some of which have been studied or are being studied. However, the functionality of most lncRNAs remains unknown. Usually, most lncRNAs exert their function through the interaction with the corresponding RNA-binding proteins. Although we have succeeded in identifying some RNA-binding proteins in the human genome and this number is growing steadily (Cook et al., 2011; Ray et al., 2013), we are not fully aware of the association between lncRNA and protein and its function in the post-transcriptional regulating network (Mittal et al., 2009; Kishore et al., 2010). Moreover, the experimental identification of lncRNA-protein associations is time-consuming, laborious and costly, so it is necessary to develop effective computational prediction methods.

At present, computational models have been broadly utilized in bioinformatics such as lncRNA-disease interactions prediction (Zeng et al., 2015; Chen et al., 2016b,d,e, 2017c,d; Huang et al., 2016; Li et al., 2016; Liu et al., 2016; Zhao et al., 2016a; Zou et al., 2016; Zhang et al., 2017a,b; Hu et al., 2018; Tang et al., 2018). However, only a few models can be used to forecast lncRNA-protein associations. For example, Bellucci et al. (2011) proposed catRAPID, which encoded the lncRNA-protein as a characteristic vector, and combined two value structures between lncRNA and protein forces, hydrogen bonding and Fan Dehua force. Later, Muppirala et al. (Muppirala et al., 2011) developed RPISeq, which utilized merely lncRNA and protein sequences, and used support vector machine (SVM) classifier (Hearst, 1998) and random forest (RF) (Liaw and Wiener, 2002) to predict the interactions between lncRNAs and proteins. Wang et al. presented a model, it utilized the same dataset of a paper by Muppirala et al. and similar data characteristics. Its theoretical basis was Naive Bias (NB) and Extended NB (ENB) classifier. In 2015, Suresh et al. proposed RPI-Pred (Suresh et al., 2015), a method on account of SVM, the sequences and structures of lncRNAs and proteins, and the high-order 3D structure characteristics of proteins are used in this method. In the same year, a method based on heterogeneous networks, called LPIHN, was proposed by Li et al. (2015). They predicted new lncRNA-protein associations by implementing a random walk with restart (RWR) on a constructed heterogeneous network. In a recent study, Ge et al. (2016) introduced a network bisection approach, named LPBNI. They carried out the resource allocation procedure in the lncRNA-protein dichotomous network to evaluate candidate proteins for each lncRNA to achieve the goal of predicting the absence of the interaction. Lately, Hu et al. (2017) advanced a semi-supervised method called LPI-ETSLP that revealed the lncRNA-protein associations. In particular, LPI-ETSLP did not require negative samples.

There are several problems with these methods, as follows: (1) Most of the models mentioned above don't use lncRNA-protein interactions data, but are trained using RNA-protein interactions data. This leads to a limitation on the ability to forecast the lncRNA-protein associations. (2) Some of the models utilize the NPInter (Yuan et al., 2014; Hao et al., 2016) database to predict the interactions between lncRNAs and proteins. Although NPInter is by far the best lncRNA-protein database, it only provides lncRNA's gene-protein interactions entries, and dose not directly provide the entries of lncRNA-protein interactions. If these models are directly investigated using lncRNA's gene-protein interactions, it will certainly affect the prediction results. (3) Finally, although the current researches and understanding of lncRNA-protein interactions are increasing, there isn't enough negative samples data yet, and it is hard to choose lncRNA and protein features. In order to solve these problems, we integrate the two methods of random walk and neighborhood regularized logistic matrix factorization to develop a new model called IRWNRLPI. The model utilizes known lncRNA-protein associations, protein similarity network and lncRNA similarity network to forecast possible lncRNA-protein associations. And unlike the traditional machine learning methods, IRWNRLPI uses semi-supervised learning to derive unknown information primarily through known associations and their similarities, so it does not need negative samples. In addition, our model provides a high level of importance for the nearest neighbors, thus avoiding noise information. We implement leave-one-out cross validation (LOOCV) on IRWNRLPI to evaluate its performance, resulting in the AUC of 0.9150, which indicates that the model has reliable performance. And the AUPR value of 0.7138 demonstrates the reliability of our model. Moreover, in the case study, we predict the lncRNA-protein associations of “Mus musculus” in view of the predicted score level, demonstrating that our method is generally effective.

## Materials and methods

### Dataset

Along with the development of bioinformatics, there are a number of public databases available for scientists to study lncRNA-protein interactions. The database NPInter includes experimental verification interactions between non-coding RNAs and other biomolecules (proteins, RNA and genomic DNA). NONCODE (Xie et al., 2014; Zhao et al., 2016b), a comprehensive annotation database, covers all types of non-coding RNA (not including tRNA and rRNA). And the database Uniprot (Consortium, 2015; Pundir et al., 2016) can provide us with protein sequences. With these databases, we can acquire the datasets we need for lncRNAs and proteins, which will help us to carry out our research better.

According to NPInter V2.0, we chiefly extract species for human lncRNA relevant items. We obtain 4870 items which are experimentally identified lncRNA-protein associations, covering 1114 lncRNAs and 96 proteins. From NONCODE 4.0, we can obtain lncRNA sequence information. From Uniprot, we can get the protein sequence information. Further, we remove proteins and lncRNAs that can't obtain sequences information. Besides, we delete those lncRNAs associated with only one protein, and those proteins that are associated with only one lncRNA. These data are low-similarity pairs and potential noise. Removing these data helps improve the performance of the model. Finally, we construct a dataset containing 4158 lncRNA-protein correlations, including 990 lncRNAs and 27 proteins.

### LncRNA-protein interaction matrix

To facilitate the description of lncRNA-protein interactions and the algorithmic model, matrix *Y* is denoted as the adjacency matrix of lncRNA-protein interactions, if lncRNA *l(i)* is connected with the protein *p(j), Y(l(i), p(j))* is 1, otherwise 0. According to sequence similarity matrix, the interactions between lncRNAs and proteins are measured. We screen the lncRNAs and proteins sequences which are inferior quality or cannot find their corresponding proteins and lncRNAs. The inferior quality refers to incomplete sequence information and repeated lncRNA and protein sequences. Finally, 4158 high quality lncRNA-protein associations are obtained.

### LncRNA sequence similarity matrix

In our work, we calculate the similarity of the lncRNA sequence according to the lncRNA sequence information. These lncRNAs sequences information is acquired from the NONCODE 4.0 database. As a result of filtering, we gain 990 credible lncRNAs sequences. The regularized Smith-Waterman algorithm (Pearson, 1991) is used to compute lncRNAs sequence similarity. Thus, the lncRNA sequence similarity matrix *LS* is built, where the empty *LS(l(i), l(j))* indicates the sequence similarity between lncRNA *l(i)* and *l(j). LS* is normalized as below:

$$LS(l\left(i\right),l\left(j\right))=\frac{sw(l\left(i\right),l\left(j\right))}{max(sw(l\left(i\right),l(i)),sw(l\left(j\right),l(j)))}$$

Where *sw*(*l*(*i*), *l*(*j*)) is the sequence similarity between lncRNA *l(i)* and *l(j)* calculated according to the Smith-Waterman algorithm.

### Protein sequence similarity matrix

We screen 27 dependable protein sequences on the basis of the lncRNA-protein network, they come from Uniprot (Consortium, 2015; Pundir et al., 2016) entirely. Similarly, protein sequence similarity can also be calculated by utilizing a regularized Smith-Waterman algorithm. Then, we can construct a protein sequence similarity matrix *PS*, in which the entity *PS(p(i), p(j))* expresses the sequence similarity between protein *p(i)* and *p(j)*. The *PS* is normalized as below:

$$PS(p\left(i\right),p\left(j\right))=\frac{sw(p\left(i\right),p\left(j\right))}{\max(sw(p\left(i\right),p(i)),sw(p\left(j\right),p(j)))}$$

Where *sw*(*p*(*i*), *p*(*j*)) is the sequence similarity between protein *p(i)* and *p(j)* calculated according to the Smith-Waterman algorithm.

### Work flow

**Figure 1:**
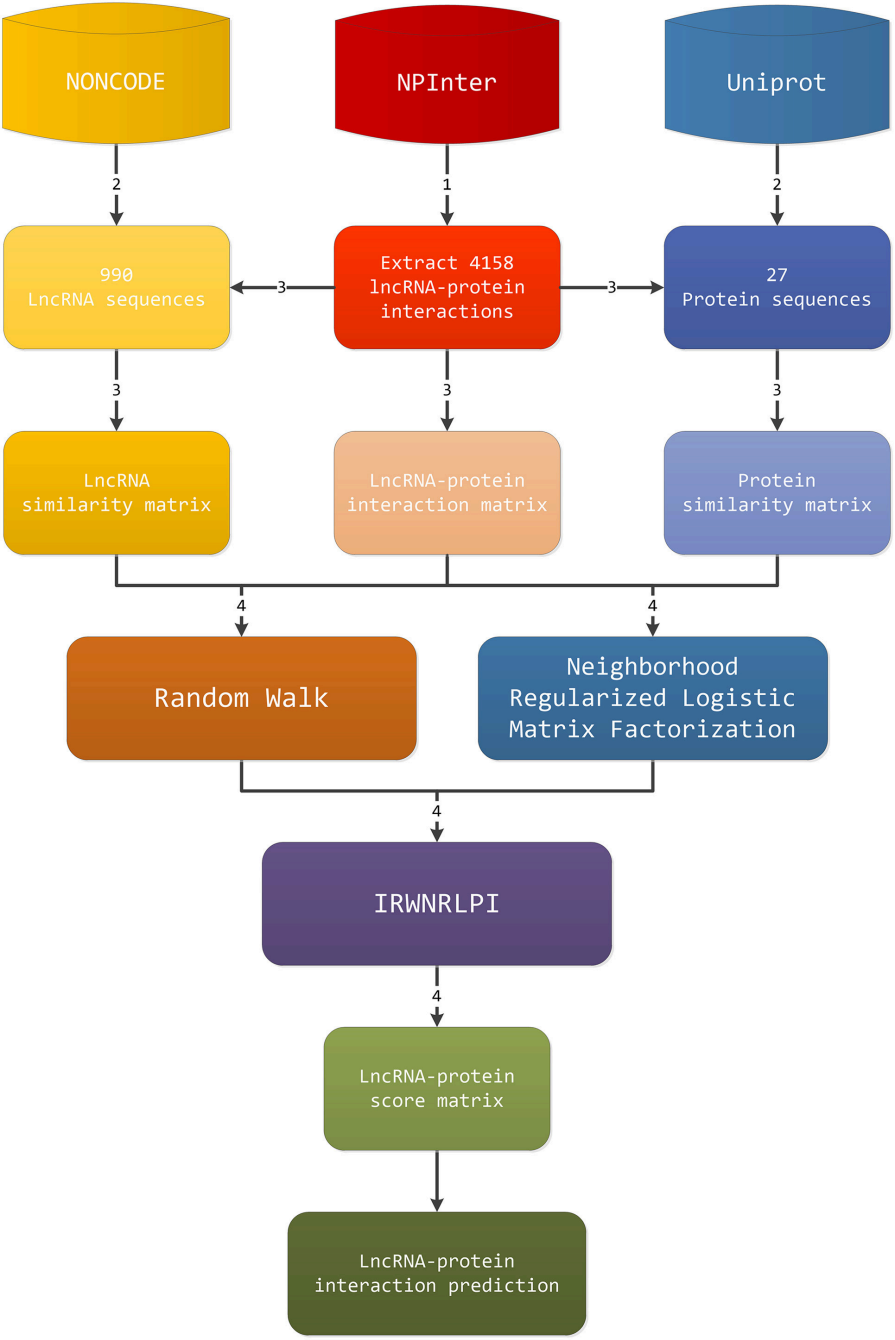
The work flow of the IRWNRLPI model.

The workflow of our IRWNRLPI model is given in Figure 1. The procedure for predicting the lncRNA-protein interactions consists of four steps. (1) Firstly, abstract gene-protein pairs information in NPInter v2.0, and we can obtain the interaction matrix between lncRNAs and proteins. (2) The second step is to extract lncRNA sequences and protein sequences from NONCODE and UniProt on account of gene-protein pairs, separately. (3) Next, we screen and remove the lncRNAs in NONCODE that fail to discovery the relevant information, as well as the protein in Uniprot that cannot seek out the corresponding information. Then, we employ the regularized Smith-Waterman algorithm to compute the similarity of lncRNA sequences and protein sequences, respectively, and generate corresponding lncRNA and protein similarity matrix. (4) Last, we will apply the three matrixes obtained above to random walk algorithm and neighborhood regularized logistic matrix factorization algorithm, respectively, to gain a potential lncRNA-protein interactions score matrix, and then enter these two score matrixes to IRWNRLPI integration model. Eventually, we gain final lncRNA-protein associations score matrix. The above is the whole prediction process to obtain new lncRNA-protein associations.

### IRWNRLPI

**Figure 2:**
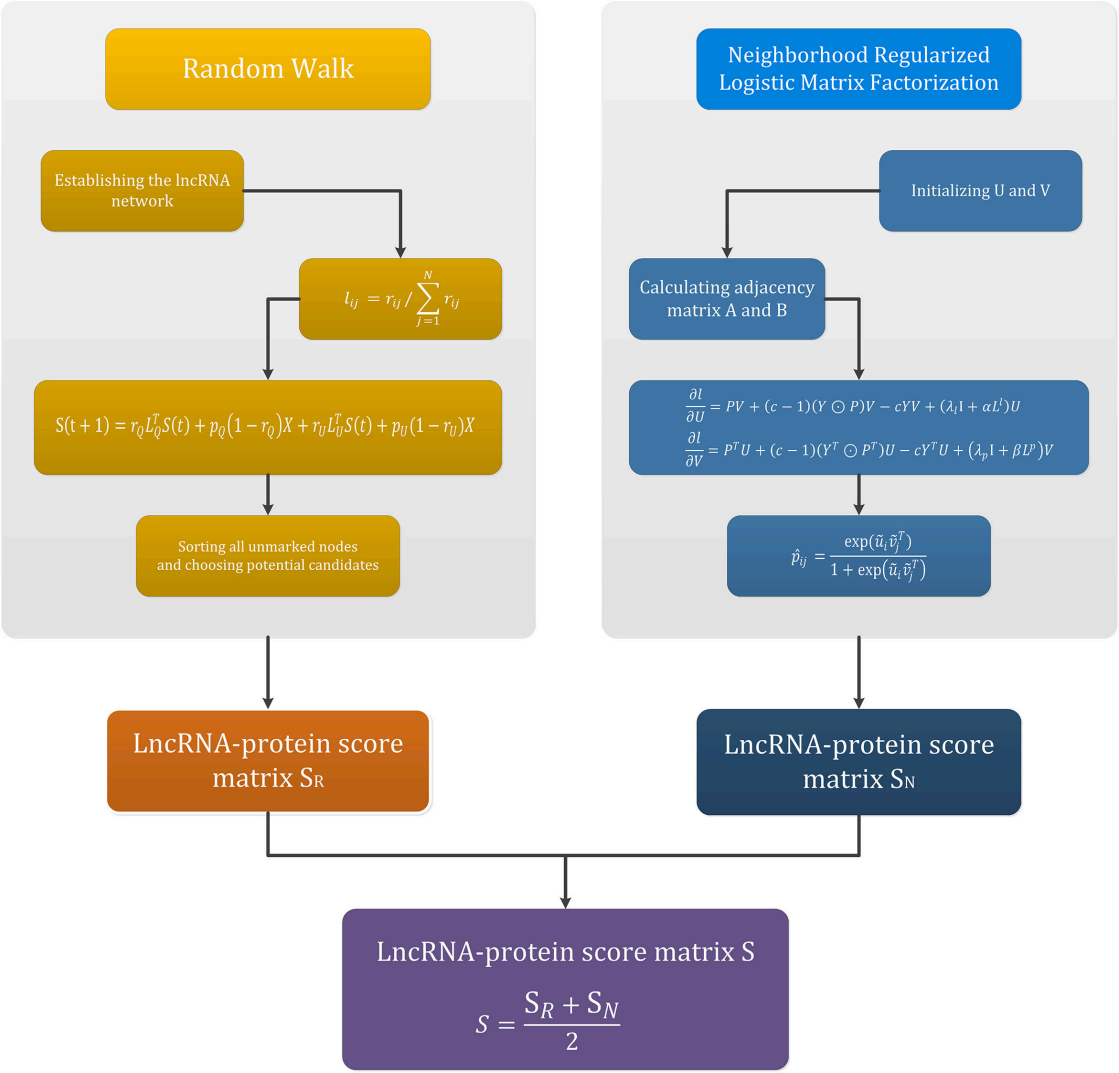
The process of constructing the integrating model.

The flowchart of this section is given in the Figure 2. The upper two parts in Figure 2 are the main flow of the random walk method and the neighborhood regularized logistic matrix factorization method, respectively. The left box is the four steps of random walk, and the lncRNA-protein score matrix *S*_*R*_ is finally obtained. The right box is the process of adjacency regularization, and finally the lncRNA-protein score matrix *S*_*N*_ is obtained. The bottom of Figure 2 is the process of obtaining the final lncRNA-protein score matrix *S* by integrating the above two methods.

#### Random walk

In random walk model, given a protein *p*, the process of predicting the lncRNAs associated with *p* is modeled as a random walk on the weighted graph G. The process can be roughly divided into four steps.

In the first step, the lncRNA network is established based on the sequence similarity between lncRNAs. For a given protein *p*, the known lncRNAs associated with *p* and the candidate lncRNAs associated with *p* and their relations form a network, expressed as a weighted graph *G* (*V, E, W*). Each vertex (*v* ∈ *V*) represents the lncRNA or candidate lncRNA associated with *p*. Each edge (*e* ∈ *E*) denotes the relationship between the two vertices connected by edge *e*. We denote sequence similarity between *v*_*x*_ and *v*_*y*_ as Sim (*v*_*x*_, *v*_*y*_), and the weight *w* of edge e is Sim (*v*_*x*_, *v*_*y*_). The greater the *w*, the more likely that the two vertices are correlated with a set of similar proteins. In this network, the known lncRNA associated with *p* is called a labeled node. The remaining lncRNAs have so far, no evidence that they are related to *p*, which are unlabeled nodes.

In the second step, constructing the correlation matrix *R* to establish two one-step transition matrices *L*_*Q*_ and *L*_*U*_. First of all, we construct the correlation matrix *R*. For *v*_*i*_, we evaluate the extent of relevance between neighbors *v*_*j*_ and *p*, which is denoted by *r*_*ij*_. Firstly, suppose that the set of all the labeled nodes is denoted as *Q*, *v*_*i*_ ∈ *Q*. If *v*_*i*_ is relevant to protein *p*, its neighbors may also be relevant to *p*. In addition, when *v*_*i*_ is a labled node, the association probability is greater than the association probability when *v*_*i*_ is an unlabeled node. Thus, the former and the latter are multiplied by *w*_*Q*_ ∈ (0,1) and *w*_*U*_ ∈ (0,1) separately. Evidently, *w*_*Q*_ is higher than *w*_*U*_. Secondly, suppose *U* is the set of all unmarked nodes, which may be associated with lncRNAs, and *v*_*i*_ ∈ *U*. If *v*_*i*_ is related to *p*, its neighbors may also be associated with *p*. The weight of the associated information from the unmarked node is *w*_*U*_. Thirdly, if the two lncRNAs are not connected, such as *v*_*i*_ and *v*_*j*_, *r*_*ij*_ is set to 0. Finally, an lncRNA to a value of itself is set to 0.

*R* (_*r*_*ij*_) *M*×*M*_ is constructed on the basis of the above rules, *r*_*ij*_ is formally defined as follows:

(1) $$r_{ij}=\left\{ \begin{array}{l} Sim\left(v_{i},v_{j}\right)\cdot w_{Q},\;v_{i}\in Q,\left(v_{i},v_{j}\right)\in E\\ Sim\left(v_{i},v_{j}\right)\cdot w_{U},\;v_{i}\in U,\left(v_{i},v_{j}\right)\in E\\ 0,\;\left(v_{i},v_{j}\right)\notin E\;or\;v_{i}=v_{j} \end{array}\right.$$

In which *v*_*i*_ is the vertex and *v*_*j*_ is one of its neighbors.

Then, we construct the transfer matrix *L* (_*l*_*ij*_) *M*×*M*_. We proportionate the transfer probability *l*_*ij*_ to *r*_*ij*_. The matrix R is normalized by the next type, and the one step transfer probability array *L* (_*l*_*ij*_) *M*×*M*_ is obtained:

(2) $$l_{ij}=r_{ij}/\sum_{j=1}^{N}r_{ij}$$

*l*_*ij*_ indicates the transition possibility from *v*_*i*_ to *v*_*j*_. Nevertheless, after the row of *R* is normalized, the weights (*w*_*Q*_ and *w*_*U*_) for distinguishing between the labeled node and the unlabeled node associated information are lost, thus ignoring the effect of the previous information about whether the vertex is relevant to *p*. In order to settle the difficulty, we divide the matrix *L* into two arrays of *L*_*Q*_ and *L*_*U*_. *L*_*Q*_ expresses the transformation array of the marked node, and *L*_*U*_ indicates the transfer matrix of the unmarked node. All lines of the marked (unmarked) node in *L*_*Q*_ (*L*_*U*_) are in accordance with the relevant rows in *L*, the rest of rows of *L*_*Q*_ (*L*_*U*_) are set to 0.

In the third step, a new forecast method on account of random walk is established to evaluate the correlation scores between each unmarked node and *p*, that is, estimate the correlation score of the candidate lncRNAs. In view of the transfer matrix *L*_*Q*_ and *L*_*U*_, the prediction method is further established as below:

(3) $$S\left(t+1\right)=r_{Q}L_{Q}^{T}S\left(t\right)+p_{Q}\left(1-r_{Q}\right)X+r_{U}L_{U}^{T}S\left(t\right)+p_{U}(1-r_{U})X$$

First, *S*(t + 1) represents a probability vector, indicating the probability that the walker reaches the *i*th vertex at time t + 1 is *S*_*i*_(*t*+1). The walker begins with the marked node, the components in *S*(0) represent the original probability, which means the walker begins at the same probability at time 0 from a marked node. And *S*_*i*_(0) calculates according to the following formula:

(4) $$S_{i}\left(0\right)=\{\begin{array}{c} \frac{1}{\left|Q\right|}\;if\;v_{i}\in Q\\ 0\;otherwise \end{array}$$

Second, to use priori information, we assign weights *r*_*Q*_ and *r*_*U*_ (0 < *r*_*Q*_, *r*_*U*_ < 1, *r*_*Q*_ > *r*_*U*_) to the labeled node and the unlabeled node, respectively. In fact, *r*_*Q*_ and *r*_*U*_ replace the ignored function of *w*_*Q*_ and *w*_*U*_. Finally, when the walker finds a marked node, at time t+1 it will go back the initial vertex (marked node) at probability *p*_*Q*_(1-*r*_*Q*_) and start walking again. The probability total of the walkers arriving at each marked node at time t is expressed as *p*_*Q*_. The formula is as follows:

(5) $$p_{Q}=\sum_{v_{i}\in Q}S_{i}(t)$$

Likewise, when the walker finds an unmarked node, at the next time it will return to the beginning vertex with possibility *p*_*U*_(1-*r*_*U*_). The probability total of the walkers arriving at each unmarked node at time t is expressed as *p*_*U*_, it is equal to 1-*p*_*Q*_. *X* defines the nodes at which the walker returns and restarts. Since walker begins with a marked node, *X* is equal to *S*(0).

The fourth step is to sort all unmarked nodes and choose potential candidates. The walker begins with the marked node and starts iterating. When the iteration satisfies the condition of convergence, the iteration procedure suspends. The convergence condition is *L*_1_-norm between *S*(t) and *S*(t + 1) less than 10^−10^. The definition of the correlation fraction of unmarked nodes is the steady state probability of the pedestrians staying at that vertex. In this way, all unmarked nodes get a correlation score, and we sort them according to their fractions. The greater the fraction, the more likely that the unlabeled node is associated with the given protein *p*. The score matrix obtain by this part is denoted by *S*_*R*_, in which *S*_*R*_*(l(i), p(j))* is the possibility of association between lncRNA *l(i)* and protein *p(j)*.

#### Neighborhood regularized logistic matrix factorization

Here we explain the neighborhood regularized logistic matrix factorization method. First, lncRNAs and proteins are mapped to shared potential spaces with dimension r, and *r* < < min (*m, n*). $u_{i}\in {\mathbb{R}}^{1\times r}$ and $v_{j}\in {\mathbb{R}}^{1\times r}$ represents the characters of lncRNA *l*_*i*_ and protein *p*_*j*_, separately. The following formula is used to calculate the probability of association *p*_*ij*_ of the lncRNA-protein pair (*l*_*i*_, *p*_*j*_):

(6) $$p_{ij}=\frac{\exp(u_{i}v_{j}^{T})}{1+\exp(u_{i}v_{j}^{T})}$$

In order to simplify, we utilize *U* ∈ ℝ^*m*×*r*^ and *V* ∈ ℝ^*n*×*r*^ to represent the set of potential vectors for all lncRNAs and all proteins.

In order to make our modeling more efficient and more accurate for lncRNA-protein interactions prediction, we recommend giving positive samples a higher level of importance than negative samples (Johnson, 2014; Liu et al., 2014), the weight of the positive sample given above is c, the weight of the negative sample is 1.

Suppose all samples are trained independently, and the probability as follows:

(7) $$\begin{array}{l} p\left(Y|U,V\right)=\left(\prod_{1<i<m,1<j<n,y_{ij}=1}{\left[p_{ij}{\;}^{y_{ij}}{\left(1-p_{ij}\right)}^{\left(1-y_{ij}\right)}\right]}^{c}\right)\\ \;\times \left(\prod_{1<i<m,1<j<n,y_{ij}=0}\left[p_{ij}{\;}^{y_{ij}}{\left(1-p_{ij}\right)}^{\left(1-y_{ij}\right)}\right]\right) \end{array}$$

Note that when *y*_*ij*_ = 1, *c*(1−*y*_*ij*_) = 1−*y*_*ij*_, when *y*_*ij*_ = 0, *cy*_*ij*_ = *y*_*ij*_. So, we rewrite the formula (7) as follows:

(8) $$\begin{array}{l} p\left(Y|U,V\right)=\left(\prod_{1<i<m,1<j<n,y_{ij}=1}p_{ij}{\;}^{cy_{ij}}{\left(1-p_{ij}\right)}^{\left(1-y_{ij}\right)}\right)\\ \;\times \left(\prod_{1<i<m,1<j<n,y_{ij}=0}p_{ij}{\;}^{cy_{ij}}{\left(1-\;p_{ij}\right)}^{\left(1-y_{ij}\right)}\right)\\ \;=\prod_{i=1}^{m}\prod_{j=1}^{n}p_{ij}{\;}^{cy_{ij}}\left(1-\;p_{ij}\right)\left(1-y_{ij}\right)\; \end{array}$$

In addition, we will carry out zero mean spherical Gaussian priori on the potential vector of lncRNA and protein:

(9) $$p\left(U|\sigma_{l}^{2}\right)=\prod_{i=1}^{m}N(u_{i}|0,\sigma_{l}^{2}I),\;p\left(V|\sigma_{p}^{2}\right)=\prod_{j=1}^{n}N(v_{j}|0,\sigma_{p}^{2}I)$$

Among them, $\sigma_{l}^{2}$ and $\sigma_{p}^{2}$ are to regulate the variance of the Gaussian distribution, I is the unitary array. So, through Bayesian inference, we have:

(10) $$p\left(U,V|Y,\sigma_{l}^{2},\sigma_{p}^{2}\right)\propto p\left(Y|U,V\right)p\left(U|\sigma_{l}^{2}\right)p\left(V|\sigma_{p}^{2}\right)$$

Thus, the posterior distribution logarithm is as below:

(11) $$\begin{array}{l} \log p\left(U,V|Y,\sigma_{l}^{2},\sigma_{p}^{2}\right)=\sum_{i=1}^{m}\sum_{j=1}^{n}cy_{ij}u_{i}v_{j}{\;}^{T}\\ \;-(1+cy_{ij}-y_{ij})\log\left[1+\;\exp\left(u_{i}v_{j}{\;}^{T}\right)\right]\\ \;-\;\frac{1}{2\sigma_{l}^{2}}\sum_{i=1}^{m}||u_{i}|{|}_{2}^{2}\\ \;-\;\frac{1\;}{2\sigma_{p}^{2}}\sum_{j=1}^{n}||v_{j}|{|}_{2}^{2}+C\; \end{array}$$

Where *C* is an absolute term. Maximizing the posterior distribution is same as minimizing the below object functions:

(12) $$\begin{array}{l} \underset{U,V}{min}\sum_{i=1}^{m}\sum_{j=1}^{n}(1+cy_{ij}-y_{ij})\log\left[1+\exp\left(u_{i}v_{j}{\;}^{T}\right)\right]\\ -cy_{ij}u_{i}v_{j}{\;}^{T}+\frac{\lambda_{l}}{2}{\left\|U\right\|}_{F}^{2}+\;\lambda_{p}2 \end{array}$$

Where, $\lambda_{l}=\frac{1}{\sigma_{l}^{2}}$ and $\lambda_{p}=\frac{1}{\sigma_{p}^{2}}\;$ and ||•||_*F*_ show the Frobenius norm of the array. Alternating gradient descent method (Johnson, 2014) can resolve the difficulty in Equation (12).

By mapping lncRNAs and proteins to shared potential space, the logistic matrix factorization method can effectually evaluate the monolithic structure of lncRNA-protein interactions information. In addition, we use lncRNAs and proteins neighbors to further advance the forecast veracity. For lncRNA *l*_*i*_, we denote the nearest neighbor set with *N*(*l*_*i*_) ∈ *L*\*l*_*i*_, where *N*(*l*_*i*_) makes up selecting the *K*_1_ most similar lncRNAs of *l*_*i*_. After that, we structure the set *N*(*p*_*j*_) ∈ *P*\*p*_*j*_, which is made up of the *K*_1_ most similar proteins of *p*_*j*_. In the experiment, we set *K*_1_ to 5 according to experience.

Here, the lncRNA neighborhood information can be represented by the adjacency array *A*, and *a*_*iμ*_ is defined as below:

(13) $$a_{i\text{μ}}=\{\begin{array}{c} s_{i\text{μ}}^{l}\;if\;l_{\text{μ}}\in N(l_{i})\\ 0\;otherwise\; \end{array}$$

The protein neighborhood information is described by the adjacency matrix *B*, and *b*_*jv*_ is defined as below:

(14) $$\;b_{jv}=\{\begin{array}{c} s_{jv}^{p}\;if\;p_{v}\in N(p_{j})\\ 0\;otherwise\; \end{array}$$

It should be noted that matrix *A* and *B* are asymmetric.

The main idea of predicting lncRNA-protein interactions with lncRNAs neighborhoods information is to minimize the distance between *l*_*i*_ and its nearest neighbor *N*(*l*_*i*_) in the potential space, which can be gained by minimizing the below object functions:

(15) $$\begin{array}{l} \frac{\alpha }{2}\sum_{i=1}^{m}\sum_{\mu =1}^{m}a_{i\text{μ}}||u_{i}-u_{\mu }|{|}_{F}^{2}=\frac{\alpha }{2}[\sum_{i=1}^{m}\left(\sum_{\mu =1}^{m}a_{i\text{μ}}\right)u_{i}u_{i}{\;}^{T}\\ \;+\sum_{\mu =1}^{m}\left(\sum_{i=1}^{m}a_{i\text{μ}}\right)u_{\mu }u_{\mu }{\;}^{T}]\\ \;\frac{\alpha }{2}tr\left(U^{T}AU\right)-\;\frac{\alpha }{2}tr\left(U^{T}A^{T}U\right)\\ \;=\;\frac{\alpha }{2}tr\left(U^{T}L^{l}U\right) \end{array}$$

Among them, tr(•) is matrix trace, $L^{l}=\left(D^{l}+{\tilde{D}}^{l}\right)-\left(A+A^{T}\right)$. *D*^*l*^ and ${\tilde{D}}^{l}$ are two diagonal arrays, where diagonal elements are ${D^{l}}_{ii}=\overset{m}{\underset{\mu =1}{\sum }}a_{i\mu }$ and ${{\tilde{D}}^{l}}_{\mu \mu }=\overset{m}{\underset{i=1}{\sum }}a_{i\mu }$ separately. We also minimize the following objective functions to use the neighborhood information of the protein for lncRNA-protein interactions prediction:

(16) $$\frac{\beta }{2}\sum_{j=1}^{n}\sum_{v=1}^{n}b_{jv}||v_{j}-v_{v}|{|}_{F}^{2}=\frac{\beta }{2}tr\left(V^{T}L^{p}V\right)\;$$

Wherein, $L^{p}=\left(D^{p}+{\tilde{D}}^{p}\right)-\left(B+B^{T}\right),\;D^{p}\;$ and ${\tilde{D}}^{p}\;$are two diagonal arrays, where diagonal elements are ${D^{p}}_{jj}=\overset{n}{\underset{v=1}{\sum }}b_{jv}$ and ${{\tilde{D}}^{p}}_{vv}=\overset{n}{\underset{j=1}{\sum }}b_{jv}$ respectively.

By taking into account lncRNA-protein associations and lncRNAs and proteins *K*_1_ the nearest neighborhoods, the final prediction model can be derived. By substituting Equations (15, 16) into Equation (12), the resulting model is as follows:

(17) $$\begin{array}{l} \underset{U,V}{min}\sum_{i=1}^{m}\sum_{j=1}^{n}\left(1\text{​}+\text{​}cy_{ij}\text{​}-\text{​}y_{ij}\right)\ln\left[1\text{​}+\text{​}\exp\left(u_{i}v_{j}{\;}^{T}\right)\right]\text{ ​}-\text{​}cy_{ij}u_{i}v_{j}{\;}^{T}\\ +\;\frac{1}{2}tr\left[U^{T}\left(\lambda_{l}I+\alpha L^{l}\right)U\right]\;+\frac{1}{2}tr\left[V^{T}\left(\lambda_{p}I+\beta L^{p}\right)V\right]. \end{array}$$

An alternating gradient rise process can resolve the optimization problem in Equation (17), which is represented as *L*, the gradient relative to *U* and *V* as below:

(18) $$\frac{\partial l}{\partial U}=PV+\left(c-1\right)\left(Y⊙P\right)V-cYV+\left(\lambda_{l}I+\alpha L^{l}\right)U$$

(19) $$\frac{\partial l}{\partial V}=P^{T}U+\left(c-1\right)\left(Y^{T}⊙P^{T}\right)U-cY^{T}U+\left(\lambda_{p}I+\beta L^{p}\right)V$$

*P* ∈ *R*^*m*×*n*^, and *p*_*ij*_ (see Equation 1) represents the Hadamard product of the two arrays. In order to quicken the constriction of the gradient decline optimization method, we utilize the AdaGrad algorithm to adaptively select the grad step length.

If potential carriers *U* and *V* are known, the association probability of any unknown lncRNA-protein pair (*l*_*i*_, *p*_*j*_) can be forecasted by formula (6). The negative dataset *L*^−^ *and P*^−^ *of lncRNAs and proteins might influence on lncRNA*−*protein interactions*. The set of *K*_2_ nearest neighbors in *L*^+^ and *P*^+^ are denoted as *N*^+^(*l*_*i*_) and *N*^+^(*p*_*j*_) for lncRNA $l_{i}\in L^{-}$ and protein $p_{j}\in P^{-}$. *N*^+^(*l*_*i*_) and *N*^+^(*p*_*j*_) are structured utilizing the same standard as utilized to structure neighborhoods during the training procedure. Then, the interaction probability between lncRNA *u*_*i*_ and protein *v*_*j*_ is modified to:

(20) $${\hat{p}}_{ij}=\frac{exp({\tilde{u}}_{i}{\tilde{v}}_{j}^{T})}{1+exp\left({\tilde{u}}_{i}{\tilde{v}}_{j}^{T}\right)},$$

where

(21) $${\tilde{u}}_{i}=\{\begin{array}{lll} & u_{i} & if\;l_{i}\in L^{+}\\ \frac{1}{\sum_{\mu \in N+\left(l_{i}\right)}s_{i\mu }^{d}} & \sum_{\mu \in N+\left(l_{i}\right)}s_{i\mu }^{d}u_{\mu } & if\;l_{i}\in L^{-} \end{array}$$

Note that Equation (21) shows a general case of smooth learning lncRNA specificity and target-specific potential carriers. In our experiment, *K*_2_ is set to 5 based on experience. The score matrix obtained by this part is denoted by *S*_*N*_, and *S*_*N*_*(l(i), p(j))* is the possibility of association between lncRNA *l(i)* and protein *p(j)*.

### Integrating model

At last, to avoid the unsatisfactory result of using one of the two methods alone, we adopt an integration strategy and propose the integration model IRWNRLPI. Here we combine the two algorithms of random walk and neighborhood regularized logistic matrix factorization, and obtain a desired result. The specific approach is that we use these two algorithms obtain two score matrix *S*_*R*_ and *S*_*N*_, and then take the average. The final fraction array is denoted as *S*, and *S(l(i), p(j))* is the possibility of association between lncRNA *l(i)* and protein *p(j)*. The formula is as follows:

(22) $$S=\frac{S_{R}+S_{N}}{2}$$

## Results

### Performance evaluation

In this work, to measure the capability of our IRWNRLP model, we perform LOOCV on lncRNA-protein interactions that have been experimentally verified. In the LOOCV experiment, it is assumed that a total of N samples, one of them is selected as a test sample, and the rest of the samples are selected as training samples. So, we result in N classifiers, N test results, and we will utilize the average of the N results to evaluate the capability of our method. Use the LOOCV to obtain the receiver operator characteristics (ROC) curve and calculate the area under ROC curve (AUC). AUC is an important popular metric for evaluating the classification model. If AUC = 1, IRWNRLPI has perfect performance; if AUC = 0.5, it represents random performance. There is also a popular indicator the area under prediction recall curve (AUPR), it is more adaptive for category unbalanced datasets because it penalizes false positives more in the assessment. Because of the presence of massive unknown labeled data in the dataset, AUPR is used to lessen the impact of misinformation for false positives on the function of the prediction model. The larger the value of AUPR, the better the capability of the method.

For adequately examining the capability of the method, we introduce the following indicators to evaluate our method: ACC (overall accuracy), SEN (sensibility), PRE (precision), and F1 (F1-scores), these indices are extensively utilized in bioinformatics, remarked as (Chen et al., 2016a, 2017a):

$$\begin{array}{l} \text{ACC}=\frac{\text{TP+TN}}{\text{TP}+\text{FP}+\text{FN}+\text{TN}}\\ \text{SEN}=\frac{\text{TP}}{\text{TP}+\text{FN}}\\ \text{PRE}=\frac{\text{TP}}{\text{TP}+\text{FP}}\\ \text{ F1}=\frac{\text{2}\times \text{TP}}{\text{2}\times \text{TP}+\text{FP}+\text{FN}}\\ \;=\text{2}\cdot \frac{\text{PRE}\cdot \text{REC}}{\text{PRE}+\text{REC}} \end{array}$$

Where TP represents true positive, TN is true negative, FP is false positive, FN is false negative. ACC is the index of systematic error, up to 100% of ACC indicates that the prediction is perfect, and in the random prediction ACC can only get 50%. Other metrics in the binary classification can also measure the capability of the method. PRE indicates the quantity of true positive predictions in the positive prediction, and SEN is also called recall, indicating the positive predictions amount of the positive samples that are properly forecasted. Considering the accuracy and sensibility of the test, the fractional value obtained by calculating the F1-score (F-score or F degree measure) can reflect if the classification model is robust. F1 is 1 for perfect method, while the worst model of F1 is 0.

### Comparison with other methods on NPInter V2.0

**Figure 3:**
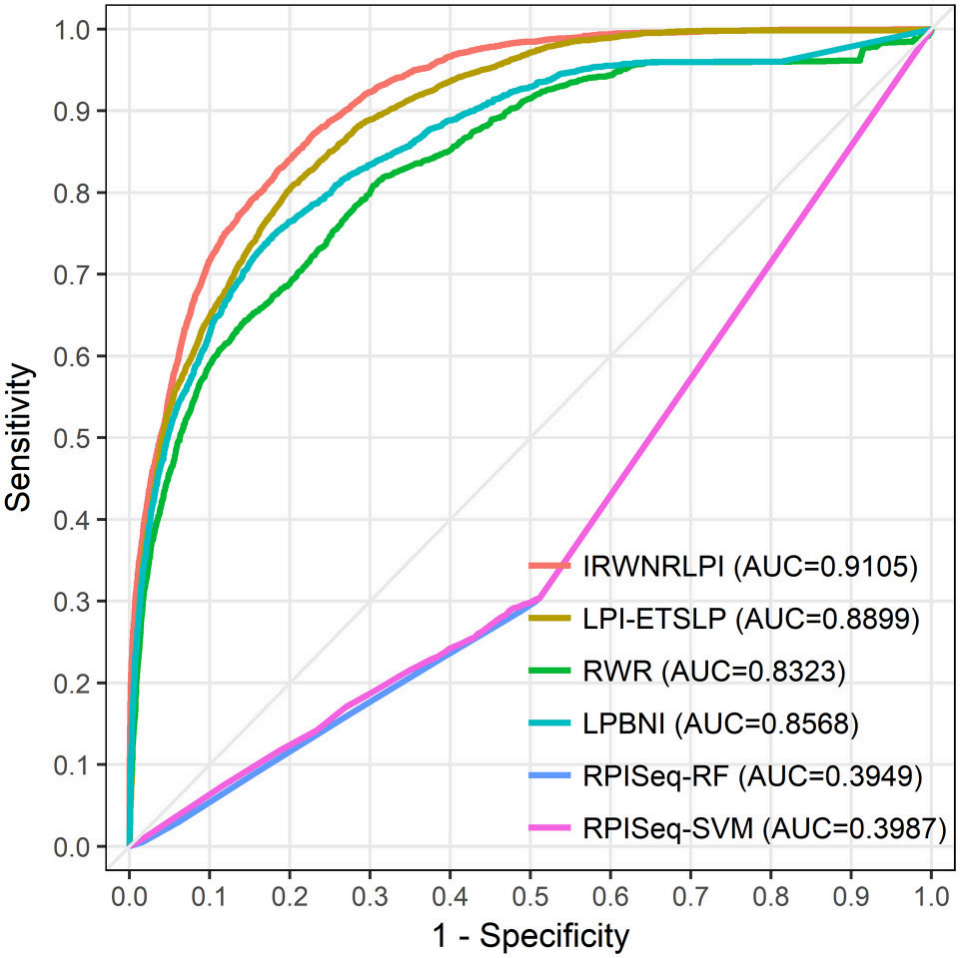
The ROC curves of IRWNRLPI, LPI-ETSLP, RWR, LPBNI, RPISeq-RF, and RPISeq-SVM are expressed in red, brown, green, blue, purple and pink, respectively. The light gray line represents the ROC curve of the interaction between IRWNRLPI and the randomized lncRNA-protein pairs.

In this part, we compare IRWNRLPI with other four models on NPInter v2.0, which are LPI-ETSLP, RWR, LPBNI, and RPISeq. Among them, RPISeq is compared with IRWNRLPI as an example of the machine learning model, in view of RF and SVM classifiers. The other three methods, LPI-ETSLP, RWR, and LPBNI, forecast potential correlations with IRWNRLPI using identical type of lncRNA and protein sequences information. The results of IRWNRLPI and the other four models are displayed in Figure 3 and Table 1, and indicate that IRWNRLPI is more ideal than the others by comparison.

**Table 1:** Comparison of IRWNRLPI with LPI-ETSLP, RWR, LPBNI, and RPISeq models.

**Methods**	**AUC**	**AUPR**	**ACC**	**PRE**	**SEN**	**F1-score**
IRWNRLPI	0.9150	0.7138	0.9009	0.7187	0.5960	0.6516
LPI-ETSLP	0.8876	0.6438	0.8834	0.5932	0.9239	0.5978
RWR	0.8332	0.2893	0.9536	0.3680	0.3538	0.3603
LPBNI	0.8586	0.3306	0.9581	0.3713	0.4139	0.3868
RPISeq-RF	0.3949	0.0631	0.4626	0.0983	0.3003	0.1481
RPISeq-SVM	0.3987	0.0698	0.4823	0.1003	0.2922	0.1493

We perform all of these models on the same dataset, and implement LOOCV experiments to compare their performance. As shown in Figure 3, our IRWNRLPI method has a AUC value of 0.9150, well above 0.5 (random), indicating that this model is feasible to predict lncRNA-protein associations. And we can see that the AUC of IRWNRLPI is higher than those of LPI-ETSLP (0.8876), RWR (0.8332), LPBNI (0.8586), RPISeq-RF (0.3949), and RPISeq-SVM (0.3987). Obviously, RPISeq is much worse than other models, even less than 0.5 (random). There are two reasons for this result: First, RPISeq is a machine learning method and depends on data, and our model does not have negative sample set; Second, RPISeq utilizes RNA-protein associations to train rather than lncRNA-protein associations, whereas the biological function of lncRNA differs from the biological function of common RNA, thus affecting the final outcome. In contrast, IRWNRLPI can avoid the problem of feature selection, thereby avoiding reliance on negative sample datasets.

From the indicators in Table 1, we can see that the prediction ability of IRWNRLPI is obviously superior to the other four methods. First, we compare the values of AUPR, which are 0.6438 (LPI-ETSLP), 0.2893 (RWR), 0.3306 (LPBNI), 0.0631 (RPISeq-RF), and 0.0698 (RPISeq-SVM) respectively. The above values are lower than 0.7138 (IRWNRLPI), indicating that the prediction result of IRWNRLPI is more dependable. Next, we further analyze the ACC, PRE, SEN, and F1-score of these models. As we can see the ACC of IRWNRLPI is less than RWR and LPBNI, owing that IRWNRLPI predicts potential lncRNA-protein associations based on known lncRNA-protein correlations, but for now, experimentally verified lncRNA-protein interactions are still less. Consequently, it is not difficult to forecast, with the lncRNA-protein associations data continuing increasing, IRWNRLPI prediction accuracy will greatly improve. In addition, it is more reasonable for this unbalanced dataset to evaluate the F1-score than using the ACC evaluation. From Table 1, it is easy to find, the F1-score of IRWNRLPI is higher than those of other methods, especially RWR and LPBNI. Our IRWNRLPI results show prediction accuracy (PRE) of 0.7187, which is approximately 21, 95, and 94% higher than LPI-ETSLP, RWR and LPBNI, separately, much higher than RPISeq-RF and RPISeq-SVM results. The sensibility (SEN) is 0.5960, it is 68, 44, 98, and 104% higher than RWR, LPBNI, RPISeq-RF, and RPISeq-SVM, separately. This results further demonstrate that IRWNRLPI performs better in forecasting lncRNA-protein associations.

### Case study

To evaluate the capability of the prediction method more comprehensively, we use IRWNRLPI to forecast potential lncRNA-protein interactions in view of the known associations of “Mus musculus” in the NPInter v3.0 dataset. The top 10 lncRNA-protein interactions are displayed in Table 2, and finally the data is centrally checked and fully verified in the “Mus musculus”. Moreover, we describe their ranking of in other methods, and it is not difficult to see from Table 2 that some of them do not get a high rank in the prediction of other models, which can lead that some new discoveries may be neglected by corresponding models. On the contrary, our model can find and confirm the interactions of these lncRNAs with proteins, and the corresponding genes are displayed in Table 2. The loss function of massive lncRNAs expressed in mouse embryonic cells is studied to show the influence on gene expression. Studies have indicated lncRNA regulates the impact of tumor cells on blood vessels, which can affect the mechanism of tumorous growth. In our forecast outcomes, NONMMUG002214-Q13185, NONMMUG013483-A2AC19 and NONMMUG015351-Q88974 are forecasted to have associations in the top 10 results of these methods, which are studied by Guttman et al. (2009). In terms of outcomes, IRWNRLPI is obviously superior in forecasting potential lncRNA-protein associations to other methods.

**Table 2:** Top 10 novel interactions predicted by IRWNRLPI and their ranks in the prediction of other methods.

**lncRNA**	**Protein**	**Confirmed?**	**IRWNRLPI**	**LPI-ETSLP**	**RWR**	**LPBNI**	**RPISeq-RF**	**RPISeq-SVM**
NONMMUG002214	Q13185	Confirmed	1	10	37	43	31	129
NONMMUT013483	A2AC19	Confirmed	2	36	39	3	178	49
NONMMUT015351	O88974	Confirmed	3	33	36	41	60	133
NONMMUT030867	Q9NQR1	Confirmed	4	37	38	2	173	137
NONMMUT045923	P83916	Confirmed	5	8	4	1	66	119
NONMMUT009968	Q8VCQ4	Confirmed	6	1	27	15	70	114
NONMMUT035343	Q9CQJ4	Confirmed	7	6	28	10	26	127
NONMMUT035346	H0YJU4	Confirmed	8	7	3	6	136	91
NONMMUT078379	Q8CGG4	Confirmed	9	17	20	12	51	144
NONMMUT040640	Q8CHK4	Confirmed	10	9	32	28	32	162

## Discussion

LncRNA involves a variety of important cellular regulatory processes and many disease progression processes, particularly in the development of various cancers. In general, most lncRNAs play their function by interacting with the corresponding RNA-binding proteins. Therefore, predicting the new lncRNA-protein associations is conducive to the research of lncRNA. Nevertheless, lncRNA-protein interactions experiments will cost a lot of materials, human and financial resources. Therefore, the utilization of computational methods to forecast lncRNA-protein associations arouses widespread concern. In our work, to obtain better prediction results, we introduce the idea of integrating algorithm and present the IRWNRLPI method, which integrates two prediction methods, random walk and neighborhood regularized logistic matrix factorization, to forecast lncRNA-protein interactions. IRWNRLPI bases only on experimentally validated lncRNA-protein associations, which avoids dependence on negative sample datasets. We conduct a more comprehensive evaluation of IRWNRLPI, test our model in the NPInter v2.0 dataset, and compare it with other four methods. In the LOOCV experiment, the AUC value of IRWNRLPI is 0.9150, indicating that IRWNRLPI performs well in the forecast of lncRNA-protein correlations. And IRWNRLPI obtains the AUPR value of 0.7138, which states clearly the responsibility of this method. In addition, we use the “Mus musculus” dataset as a case study to test IRWNRLPI and investigate the practical capability of this method in forecasting unknown lncRNA-protein associations. Case study shows that IRWNRLPI is able to forecast other new lncRNA-protein interactions. With the continuous progress of science and technology, more and more lncRNA-protein interactions will be found, and then the accuracy of IRWNRLPI prediction will also increase. In conclusion, IRWNRLPI is an efficient model of predicting potential lncRNA-protein associations, and we also hope that IRWNRLPI can be used in a wider range of studies.

The excellent and reliable predictive performance of IRWNRLPI is mainly attributable to the following factors. Firstly, unlike the traditional machine learning methods, IRWNRLPI uses semi-supervised learning to derive unknown information primarily through known associations and their similarities, so it does not need negative samples. Secondly, our model provides a high level of importance for the nearest neighbors, thus avoiding noise information. Thirdly, IRWNRLPI is a model based on an integrated idea, and the integration model gets better results than a single model.

Of course, IRWNRLPI also needs to be improved for the following reasons. First of all, the proposed model relies heavily on the known correlation data, but the number of current known lncRNA-protein associations is still very limited. As the number of experimentally validated associations increasing in future, the prediction accuracy of our method will improve. Furthermore, when the training sample changes, the prediction effect will be unstable. In addition, further consideration should be given on how to choose the value of the model parameters more properly.

## Author contributions

QZ and HL conceived the project, developed the prediction method, designed and implemented the experiments, analyzed the result, and wrote the paper. YZ implemented the experiments, analyzed the result, and wrote the paper. HH, GR, and WZ analyzed the result. All authors read and approved the final manuscript.

### Conflict of interest statement

The authors declare that the research was conducted in the absence of any commercial or financial relationships that could be construed as a potential conflict of interest.
